# The relationship between maternal vitamin D levels and osteopenia development in preterm infants: A cross-sectional study

**DOI:** 10.18502/ijrm.v22i11.17818

**Published:** 2025-01-10

**Authors:** Razieh Sadat Tabatabaei, Seyed Reza Mirjalili, Atefeh Ashrafi, Farimah Shamsi

**Affiliations:** ^1^Department of Obstetrics and Gynecology, School of Medicine, Shahid Sadoughi University of Medical Sciences, Yazd, Iran.; ^2^Mother and Newborn Health Research Center, Shahid Sadoughi University of Medical Sciences, Yazd, Iran.; ^3^Department of Obstetrics and Gynecology, Shahid Beheshti Hospital, Esfahan University of Medical Sciences, Ardestan, Iran.; ^4^Department of Biostatistics and Epidemiology, School of Public Health, Shahid Sadoughi University of Medical Sciences, Yazd, Iran.

**Keywords:** Vitamin D, Osteopenia, Prematurity, Alkaline phosphatase.

## Abstract

**Background:**

Osteopenia of prematurity (OP) is characterized by reduced bone mineral content, and vitamin D deficiency may worsen OP by affecting bone metabolism.

**Objective:**

This study aimed to investigate the correlation between maternal vitamin D levels and biochemical markers related to OP.

**Materials and Methods:**

This analytical cross-sectional study, conducted at Shahid Sadoughi hospital, Yazd, Iran, from June 2022 to September 2023, included 49 pregnant women and their preterm infants. Based on the serum alkaline phosphatase and phosphorus levels of the infants at birth and 4 wk after birth, they were divided into osteopenic and non-osteopenic groups. Maternal and neonatal vitamin D and calcium levels were then compared between these groups.

**Results:**

The mean maternal vitamin D level was 24.9 ± 15.09 ng/ml, with 36% showing deficiency. 11 neonates at 4 wk exhibited osteopenia based on phosphorus and serum alkaline phosphatase levels. Those with osteopenia had significantly lower vitamin D (p = 0.032) and calcium levels compared to non-osteopenic neonates (p = 0.043), although maternal vitamin D mean was not a significant risk factor for OP (p = 0.313).

**Conclusion:**

The results suggest that maternal vitamin D levels do not have a significant association with the incidence of osteopenia in neonates, as the mean maternal vitamin D concentration was not identified as a risk factor for this condition. Therefore, it is recommended that future research should investigate alternative factors that may contribute to the development of osteopenia in preterm infants.

## 1. Introduction

Premature neonates are at risk of developing osteopenia, a condition marked by reduced mineralization of the skeletal system compared to those who reach full term in the womb (1). This typically occurs 3–12 months after birth, with a prevalence of up to 60% in extremely low birth weight neonates and 20% in very low birth weight neonates (2). Moreover, evidence has shown defined rickets in 10–20% of preterm neonates with birth weights ≤ 1000 gr (3).

Risk factors for osteopenia of prematurity (OP) include low gestational age (GA) and birth weight, extreme prematurity, maternal chorioamnionitis, prolonged total parenteral nutrition, use of corticosteroids, methylxanthines, diuretics, immobilization, and sedation (4–6). Placental calcium and phosphate transfer to the fetus significantly rise after 24 wk of gestation. Inadequate calcium and phosphorus intake post-birth or through parenteral nutrition can lead to bone deformities and fractures in neonates (7–9). Additionally, factors such as lack of phosphate intake, prolonged parenteral nutrition, high alkaline phosphatase (ALP) levels, onset of vitamin D supplementation, and premature rupture of membranes are associated with the incidence of OP (7).

ALP is a commonly used marker for OP. Studies have shown that elevated ALP levels are linked to osteopenia occurrence in neonates. Serum calcium and phosphorus levels have also been studied as markers for OP (4, 10–12). Low serum inorganic phosphate levels have been significantly associated with osteopenia in premature neonates (11). Other markers such as parathyroid hormone (PTH) and vitamin D concentrations have also been investigated (6). Maternal or neonatal vitamin D deficiency can exacerbate osteopenia in preterm and very low birth weight neonates by affecting calcium and bone metabolism (9, 13).

Thus, this study aimed to establish the occurrence of osteopenia in premature neonates and examine the influence of maternal vitamin D on their bone health.

## 2. Materials and Methods

### Participants 

In this cross-sectional study, data of 49 pregnant women with premature labor and their neonates, referred to the gynecology and neonatology wards, Shahid Sadoughi hospital, Yazd, Iran, from June 2022 to September 2023, was extracted from their medical records.

The inclusion criteria were 18 and 33 yr pregnant women without maternal chorioamnionitis who experienced preterm labor (GA ≤ 34 wk based on the last menstrual period and ultrasound) and took 1000 units of vitamin D supplements daily until delivery, who has their first delivery.

Moreover, exclusion criteria were GA > 34 wk, congenital skeletal anomalies, multiple gestations, and refusal of follow-up visits.

Data on maternal and neonatal demographics, and clinical status including: complications, GA, delivery type, neonate gender, medication use, mechanical ventilation, feeding status, and hospitalization duration were extracted from newborn medical records. Routine laboratory tests included measuring serum vitamin D levels in mothers and preterm neonates at birth and 4 ${\;}^{\text{th}}$ wk were conducted at Shahid Sadoughi hospital, Yazd, Iran. Diagnostic biomarkers for osteopenia, including serum phosphorus, calcium, and ALP levels were also recorded. Neonates were classified into 2 groups as healthy (non-osteopenic n = 38) or at risk for osteopenia (Osteopenic n = 11) based on established biochemical marker cut-off points. Serum vitamin D and calcium levels were compared between the 2 groups to investigate potential relationships with osteopenia.

### Sample size

The sample size was estimated to be a minimum of 49 participants by considering a 95% confidence level, a power of 80%, and a standard deviation of 6.8 for the vitamin D level in mothers of infants with OP. A bias of 2 units in the estimations was also considered. The calculation utilized PASS15 software (PASS 15 Power Analysis and Sample Size Software, 2017, NCSS, LLC, Kaysville, Utah, USA, ncss.com/software/pass) and followed the following formula: 


$$n=\frac{({Z_{1-\frac{\alpha }{2}}+Z_{1-\beta })}^{2}\times S^{2}}{d^{2}}=49$$

### Biochemical analysis 

All participating mothers received a daily 1000 unit vitamin D supplement under the supervision of a gynecologist until delivery. Following delivery, a 5 ml venous blood sample was obtained to assess maternal serum 25-(OH)-vitamin D3 (Vitamin D) levels using the enzyme-linked immunosorbent assay method. Moreover, 3 ml of umbilical cord blood was collected for neonatal serum vitamin D analysis. Subsequently, all neonates were administered total parenteral nutrition therapy in the 1 ${\;}^{\text{st}}$ wk, which included calcium gluconate, glycophos, and 7000 units of vitamin D per week. To evaluate blood biochemical markers related to OP, serum calcium, ALP, and phosphorus levels were measured at birth and again at 4 wk of age using the spectrophotometric method. Based on the manufacturer's recommendation, serum vitamin D levels are categorized as deficiency ( < 20 ng/ml), insufficiency (20–30 ng/ml), and sufficiency ( > 30 ng/ml). Additionally, the cut-off points for P < 4 mmol/L or ALP > 900 IU/L were designated as predictive values for OP (14).

### Ethical Considerations

The study protocol was approved by the Ethics Committee of the Institutional Review Board of Shahid Sadoughi University of Medical Sciences, Yazd, Iran (Code: IR.SSU.MEDICINE.REC.1400.381). Before enrollment, written informed consent was obtained from all participants to use their data. Participants privacy and confidentiality were maintained, and they were not subjected to any additional costs.

### Statistical Analysis

The data analysis was performed using SPSS 26.0, a software developed by SPSS Inc. in Chicago, Illinois, USA. Quantitative variables were presented as mean ± SD, while qualitative variables were presented as numbers (percent). To establish relationships between biomarker values for OP, vitamin D concentration, and other variables, statistical tests such as independent Student *t* test, Chi-square test, and Fisher's exact tests were used. The predetermined level of significance was p < 0.05.

## 3. Results 

Of the 57 infants, 8 infants were excluded due to incomplete data, congenital skeletal anomalies, and twins, resulting in 49 neonates selected for the study. They had almost identical gender distribution and GA of 27–33^+6^ wk, with most delivered via cesarean section (8.1% vaginally). Only one mother experienced pre-eclampsia. Among the neonates, 14 (28%) faced complications, including sepsis, intrauterine growth restriction, and neurological issues. After birth, the majority received a caffeine regimen and were on “non-per oral” status, with breast milk initiated for all. Clinical characteristics are summarized in table I. Neurologic disease occurred in 2.6% of non-osteopenic and 18.2% of osteopenic infants, with no significant difference. Sepsis rates were comparable: 10.3% in non-osteopenic and 9.1% in osteopenic infants (p = 1.00). Intrauterine growth restriction was noted in 15.8% of non-osteopenic infants, with none of the osteopenic infants affected (p = 0.315). Kangaroo mother care was provided to 84.2% of non-osteopenic and 90.9% of osteopenic infants (p = 1.000), showing no association. Caffeine was given to 94.6% of non-osteopenic and all osteopenic neonates (p = 1.00). All subjects were non-per oral and mechanically intubated. High rates of breastfeeding initiation were observed in both groups (94.7% non-osteopenic vs. 100% osteopenic; p = 1.00), while breast milk fortification was rare (5.3% non-osteopenic vs. 0% osteopenic). Rehabilitation was utilized by 26.3% of non-osteopenic and 45.5% of osteopenic infants (p = 0.275), with no statistical significance.

A total of 40 neonates (81.6%) were found to have vitamin D deficiency at birth, which decreased to 14.28% 4 wk after birth. Among the neonates without osteopenia, 76.32% had vitamin D deficiency at birth. In contrast, 100% of those with osteopenia showed vitamin D deficiency at birth. However, this difference was not statistically significant (p = 0.203). By week 4, vitamin D deficiency was observed in 10.5% of the non-osteopenic group compared to 27.3% of the osteopenic group, but this difference was not statistically significant (p = 0.091).

At birth, only 2.63% of neonates without osteopenia had phosphorus levels below 4 mg/dL, compared to 45.45% of those with osteopenia (p = 0.001). By 4 ${\;}^{\text{th}}$ wk, 10.53% of non-osteopenic neonates had phosphorus levels below 4 mg/dL, while all osteopenic neonates did (p < 0.001). For ALP levels, all neonates had levels below 900 IU/L at birth. However, by 4 wk, 15.79% of non-osteopenic neonates had levels above 900 IU/L, whereas all osteopenic neonates exceeded this level (p < 0.001) (Table II).

The mean level of vitamin D in mothers was 24.9 ± 15.09 ng/ml. Neonates had vitamin D levels of 16.44 ± 5.80 ng/ml at birth, which increased to 28.45 ± 7.70 ng/ml at 4 wk old. Among the mothers, 36% were vitamin D deficient, and 40% had insufficient levels. At 4 ${\;}^{\text{th}}$ wk, neonates whose mothers had insufficient vitamin D levels faced odds of osteopenia that were 12 times higher compared to those with adequate levels (OR = 12, p = 0.053). Table III shows the vitamin D levels for both groups (determined by ALP and phosphorus cut-off points) for mothers and neonates. Interestingly, mothers of neonates with osteopenia had higher mean vitamin D levels compared to those whose neonates did not have osteopenia, though this difference was not statistically significant (p = 0.313). At birth, neonates in the osteopenia group had significantly lower vitamin D levels than those without osteopenia (p = 0.002). Furthermore, in 4 ${\;}^{\text{th}}$ wk, neonates without osteopenia exhibited higher vitamin D levels compared to those with osteopenia (p = 0.032), suggesting a trend toward better vitamin D status in the non-osteopenic group.

Table IV shows significant disparities in vitamin D and calcium levels between the 2 groups, highlighting their potential effects on neonatal health. Maternal vitamin D levels were similar across groups, with non-osteopenic levels at 23.09 ± 13.16 compared to 27.53 ± 18.28 in osteopenic neonates (p = 0.674 for ALP). Both phosphorus levels and overall comparisons reflected similar patterns. However, for 4-wk-old neonates, non-osteopenic infants had higher vitamin D levels (30.93 ± 8.18) than osteopenic ones (23.79 ± 4.00, p = 0.001 for ALP). Calcium levels also showed notable differences; non-osteopenic neonates had a mean level of 9.09 ± 0.85, while osteopenic neonates had lower levels at 8.64 ± 0.60 (p = 0.059 for ALP).

**Table 1:** Clinical characteristics of neonates in groups

**Variables**		**Osteopenic group (n = 11)**	**Non-osteopenic group (n = 38)**	**P-value**
**Gestational age**		31.91 ± 2.16	32.64 ± 1.47	0.39*
**Gender**				
	**Boy**	7 (63.6)	18 (47.6)	
	**Girl**	4 (36.4)	20 (52.4)	0.342**
**Delivery type**				
	**Vaginal**	2	4	
	**Cesarian**	9	34	0.774**
**Perinatal complications**				
	**Neurologic issues**	2 (18.2)	1 (2.6)	0.122**
	**Sepsis**	1 (9.1)	4 (10.3)	1.000**
	**IUGR**	0 (0)	6 (15.8)	0.315**
	**Kangaroo mother care**	10 (90.9)	32 (84.2)	1.000***
	**Caffeine administration**	11 (100.0)	35 (94.6)	1.000***
**Nutritional status**				
	**Initiation of breastfeeding**	11 (100.0)	36 (94.7)	
	**Initiation of breast milk fortification**	0	2 (5.3)	1.000**
**Needs to rehabilitation**		5 (45.5)	10 (26.3)		0.275***
*Data presented as Mean ± SD, Mann-Whitney test. **Data presented as n (%), Fisher's exact test, ***Data presented as n (%), Chi-square test. IUGR: Intrauterine growth restriction				

**Table 2:** Comparison of serum vitamin D and diagnostic biomarkers for osteopenia in neonates at birth and 4 wk after birth between 2 study group

**Variables**		**Osteopenic group (n = 11)**	**Non-osteopenic group (n = 38)**	**P-value**
**Vitamin D status at birth (ng/mL)**				
	**Deficient (< 20)**	11 (100)	29 (76.32)	
	**Insufficient (20–30)**	0	8 (21.05)	
	**Sufficient (30–100)**	0	1 (2.63)	0.203
**Vitamin D status at the age of 4 wk (ng/mL)**				
	**Deficient (< 20)**	3 (27.3)	4 (10.5)		
	**Insufficient (20–30)**	7 (63.6)	18 (47.4)	
	**Sufficient (30–100)**	1 (9.1)	16 (42.1)	0.091
**Phosphorus level at birth**				
	**< 4 mg/dL**	5 (45.45)	1 (2.63)		
	**> 4 mg/dL**	6 (54.55)	37 (97.37)	0.001
**Phosphorus level at the age of 4 wk**				
	**< 4 mg/dL**	11 (100)	4 (10.53)	
	**> 4 mg/dL**	0	34 (89.47)	< 0.001
**Alkaline phosphatase level at birth**				
	**< 900 IU/L**	11 (100)	38 (100)	
	**> 900 IU/L**	0	0	-
**Alkaline phosphatase level at the age of 4 wk**				
	**< 900 IU/L**	0	32 (84.21)	
	**> 900 IU/L**	11 (100)	6 (15.79)	< 0.001
Data presented as n (%), Fisher's exact test				

**Table 3:** Comparison ofmaternal and neonatal vitamin D levels between 2 study groups

**Vitamin D level (ng/ml)**	**Osteopenic group (n = 11)**	**Non-osteopenic group (n = 38)**	**P-value**
**Mothers**	31.64 ± 20.90	22.61 ± 12.59	0.313
**Neonates at birth**	11.73 ± 5.05	17.81 ± 5.42	0.002
**Neonate at the age of 4 wk**	24.12 ± 4.4	29.71 ± 8.10	0.032
Data presented as Mean ± SD, Mann-Whitney test			

**Table 4:** Comparison of serum vitamin D and calcium levels cut-off points between non-osteopenic (n = 32) and osteopenic (n = 17) groups

	**Alkaline phosphatase**		**Phosphorus**		**Both**	
**Variables**	**Non-osteopenic group**	**Osteopenic group**	**Non-osteopenic group**	**Osteopenic group**	**Non-osteopenic group**	**Osteopenic group**
		23.09 ± 13.16 (21, 10)	27.53 ± 18.28 (20, 20)	22.65 ± 13.21 (20, 10)	29.13 ± 18.39 (25, 19)	22.61 ± 12.59 (20.50, 10)	31.64 ± 20.90 (25, 41)
** Vitamin D level in**
	mothers*	p = 0.674		p = 0.282		p = 0.313	
	30.93 ± 8.18	23.79 ± 4.00	29.02 ± 7.51	27.15 ± 8.44	29.71 ± 8.10	24.12 ± 4.46
** Mean vitamin D**
	level in 4-wk-old
neonates**	p = 0.001		p = 0.443		p = 0.032	
	9.09 ± 0.85	8.64 ± 0.60	9.13 ± 0.81	8.49 ± 0.58	9.06 ± 0.80	8.51 ± 0.680
** Calcium level in**
	4-wk-old neonates**	p = 0.059		p = 0.009		p = 0.043	
*Data presented as Mean ± SD (median, interquartile range), Mann-Whitney test. **Data presented as Mean ± SD, Mann-Whitney test						

## 4. Discussion

The current study examined the effect of maternal vitamin D status on OP by measuring phosphorus and ALP levels as biomarkers. Among the 49 neonates, 11 (22%) showed signs of osteopenia at the age of 4 wk, which aligns with the reported prevalence of OP in low birth weight neonates, typically between 23% and 32% (11). Osteopenia in this study was defined by phosphorus levels below 4 mmol/L and ALP levels above 900 IU/L at 4 wk. ALP production in neonatal bone tissue increases due to limited mineral reserves, peaking during the first 3 wk of life before stabilizing at 5–6 wk (15). Contributing factors to preterm neonates' vulnerability to hypophosphatemia (phosphorus level < 4 mg/dL) and mineral bone disorders include inadequate phosphorus intake, gastrointestinal immaturity, reliance on parenteral nutrition, and the limited solubility of phosphorus (14).

Our study indicates that the incidence of hypophosphatemia increases from 12.24% at birth to 30.61% by the 4 ${\;}^{\text{th}}$ wk of life. Notably, elevated ALP levels were not observed in neonates immediately after birth; however, by 4 wk, 34% of participants had ALP levels over 900 IU/L. These findings support a comprehensive approach that considers both serum biomarkers and assessments at 4 wk of age. This strategy may be more effective in identifying at-risk cases of osteopenia than relying on a single biochemical marker or measurement at birth. Previous research has identified a combination of high ALP levels and low phosphate concentrations as predictive factors for metabolic bone disease of prematurity (MBDP) (16, 17). Supportive evidence highlights a sensitivity of 100% and specificity > 70% when using cut-off values for phosphorus level < 4 and ALP > 900 IU/L at 3 ${\;}^{\text{rd}}$ wk of age to diagnose MBDP (9). In another study, 61% of premature infants experienced hypophosphatemia in the early days of life, with 45% of these infants showing a subsequent decrease in phosphate levels (18). Furthermore, it is reported that 37% of preterm neonates exhibited metabolic bone disease potentially linked to hypophosphatemia by 4 ${\;}^{\text{th}}$ wk of age. A notably high prevalence of hypophosphatemia (91%) was documented in premature neonates receiving intensive parenteral nutrition, with an average phosphorus level of 2.52 mg/dL (19, 20). Various studies have explored different diagnostic methods and cut-off values for biochemical markers. One study utilized cut-off values of ALP > 900 IU/L and phosphorus level < 5.5 mmol/L for screening MBDP in neonates aged between 3 and 5 wk (17). Another research found that combining serum ALP > 900 IU/L and phosphorus level < 1.8 mmol/L yielded a sensitivity of 100% and specificity of 70% for screening MBDP (16). Additionally, ALP levels around 800 IU/L can indicate bone metabolic disorders (15), emphasizing the importance of evaluating ALP alongside phosphorus concentrations to improve screening sensitivity. An ALP level of 344 IU/L has been identified as the most effective predictive value for MBDP among preterm cases for those aged between 3 and 30 days (21). Moreover, MBDP has been defined using criteria of ALP > 450 IU/L and phosphorus level < 4 mg/dL or through radiographic findings in neonates aged 4 wk or younger, specifically for those born at a GA of ≤ 32 wk and a birth weight of ≤ 1500 gr (11). A study involving 120 preterm and low-birth-weight neonates assessed serum ALP levels alongside wrist or knee radiographic findings to diagnose osteopenia, identifying an ALP cut-off value of 500 IU/L as indicative of the condition (22). Overall, these findings underscore the prevalence of hypophosphatemia in preterm neonates, which may persist or worsen during the early weeks of life.

In our study, using the ALP cut-off criterion, we found that serum vitamin D and calcium levels at the age of 4 wk were significantly lower in osteopenic neonates compared to their non-osteopenic counterparts. This suggests that measuring calcium and vitamin D concentrations could serve as a convenient and effective method for the rapid diagnosis of MBDP in preterm neonates. Furthermore, the early initiation of vitamin D supplementation for at-risk neonates may enhance their outcomes (23). The relationship between osteopenia and low serum calcium levels may stem from the stimulation of PTH in preterm neonates. PTH regulates blood calcium levels, potentially at the expense of bone demineralization (24). A study by Alizadeh Taheri et al. from Iran concluded that a low dose of vitamin D (200 IU/day) effectively prevents osteopenia (25). Additionally, another study found a weak positive correlation between serum 25-hydroxy vitamin D levels and serum ionized calcium and phosphorus levels, along with a negative correlation with ALP levels (26). A case-control study indicated that higher calcium intake increased the likelihood of osteopenia, while having a partner and a shorter duration of sedative use were identified as protective factors (4). Consistent with these results, Chauhan et al. found an inverse correlation between serum calcium and ALP levels in 27 preterm neonates (28–36 wk). They also observed a positive relationship between calcium and phosphorus levels within the study population (27). A prospective observational cohort study reported no significant difference in bone mineral density or content between neonates with and without osteopenia, despite both groups receiving recommended vitamin D supplementation (28). Another study indicated a correlation between MBDP and low vitamin D and calcium levels assessed at the 3 ${\;}^{\text{rd}}$ wk of life, although it was confirmed that these factors alone could not serve as specific biochemical markers of osteopenia (29). Ercan et al. also showed lower serum vitamin D levels in neonates with craniotabes (a form of MBDP) compared to healthy counterparts (30). Conversely, an investigation found no significant difference in serum calcium levels between 2 preterm groups (27–32 wk) with and without osteopenia at the age of 3 wk (7). Overall, these studies suggest that while vitamin D and calcium levels may influence the development of osteopenia, further research is required to fully understand the connection.

Neonatal vitamin D storage at birth is dependent on maternal levels, and a deficiency can lead to bone disease in premature neonates, particularly with an incidence of up to 55% in those weighing ≤ 1000 gr. Low vitamin D status during the first trimester of pregnancy is also a risk factor for neonatal intensive care unit admission (31). However, our research findings revealed no significant correlation between maternal vitamin D levels and the incidence of osteopenia. Saleem et al. did not find notable differences in maternal vitamin D levels between groups with and without biochemical evidence of osteopenia (29.80 vs. 28.12 ng/ml; p = 0.073) (32). Similarly, Alizadeh Taheri et al. demonstrated no significant relationship between maternal vitamin D status and OP prevalence (25). However, other studies have indicated a significant association between maternal vitamin D insufficiency and MBDP during the early postnatal period (33). Discrepancies in these findings may arise from differences in the demographic and clinical characteristics of the studied populations, such as GA, criteria for defining vitamin D statuses, methods used, biochemical marker values indicating osteopenia, and sample sizes.

In our current study, no statistically significant relationships were observed between the cut-off points of biochemical markers and other neonatal variables such as: gender, non-per oral period, durations of neonatal intensive care unit admission, intubation period, or intrauterine growth restriction. To strengthen future investigations, larger sample sizes are needed. One strength of this study is the consideration of ALP alongside phosphorus and the assessment of both biochemical markers at 4 wk of age, which may help identify more cases at risk for osteopenia.

### Strengths and limitations

The study boasts several strengths, including extensive data collection on clinical and biochemical parameters in neonates, highlighting the prevalence of osteopenia and vitamin D deficiency. Results are organized based on osteopenia presence, facilitating clear comparisons. It emphasizes the importance of vitamin D for bone health and the impact of maternal health on neonatal outcomes. Additionally, vitamin D levels were measured at birth and again at 4 wk, adding a temporal aspect to the analysis. However, the study has significant limitations. The small sample size of 49 neonates may limit the generalizability of the findings and reduce the power of statistical tests, hindering definitive conclusions. Incomplete data management and lack of cooperation from mothers may introduce biases affecting validity. Moreover, potential confounding factors, such as socioeconomic status and maternal diet, are not addressed, which could influence vitamin D levels and outcomes. A 4-wk follow-up may be insufficient for assessing the long-term effects of vitamin D levels and osteopenia. Finally, redundant reporting of p-value and statistical comparisons in both text and tables may obscure key findings and lead to misinterpretations. The primary focus on biochemical markers also restricts understanding of long-term clinical outcomes related to osteopenia or vitamin D deficiency in neonates.

## 5. Conclusion

The study highlights that maternal vitamin D levels do not have a significant correlation with the incidence of osteopenia in preterm infants. While neonates exhibit low vitamin D levels at birth, these levels improve notably by 4 wk, yet many continue to show osteopenia characteristics, as indicated by low ALP and phosphorus levels. Infants diagnosed with osteopenia presented lower serum concentrations of both vitamin D and calcium, underscoring the importance of these nutrients for bone health. Despite the significant differences in vitamin D and calcium levels which could inform the identification and management of osteopenia, this research suggests that maternal vitamin D status does not directly affect the development of this condition in preterm infants. Therefore, it is evident that additional factors beyond maternal vitamin D are influential in determining bone health outcomes. The study emphasizes the necessity of regular monitoring of vitamin D, ALP, and phosphorus levels in preterm infants to evaluate and potentially reduce the risk of osteopenia. Future research should focus on examining other maternal and neonatal factors that may impact bone health, which could lead to a deeper understanding of this issue and ultimately improve management strategies for preterm infants.

##  Data Availability

The data that supports the findings of this study are available from the corresponding author upon reasonable request.

##  Author Contributions

RS. Tabatabaie and SR. Mirjalili designed and conducted the research. A. Ashrafi and SR. Mirjalili monitored and evaluated the findings, while F. Shamsi analyzed the results. All authors reviewed and approved the final manuscript and take responsibility for the integrity of the data.

##  Acknowledgments

The work was extracted from a thesis by Atefeh Ashrafi for obtaining a degree in obstetrics and gynecology. We sincerely thank all mothers who participated in this study. This study was financially supported by Shahid Sadoughi University of Medical Sciences, Yazd, Iran (Thesis number: 12126). We confirm that we did not use AI in this context and provide information and support independently, without AI influence.

##  Conflict of Interest

The authors conducted this research transparently and have no financial or personal connections that could influence the work presented in this manuscript.
